# Circulating non-coding RNAs in recurrent and metastatic ovarian cancer

**DOI:** 10.20517/cdr.2019.51

**Published:** 2019-09-19

**Authors:** Heidi Schwarzenbach, Peter B. Gahan

**Affiliations:** ^1^Department of Tumor Biology, University Medical Center Hamburg-Eppendorf, Hamburg 20246, Germany.; ^2^Fondazione “Enrico Puccinelli” Onlus, Perugia 06123, Italy.

**Keywords:** Ovarian cancer recurrence, metastasis, epithelial-mesemchmal transition, non-coding RNAs, chemotherapy, tumor markers

## Abstract

Ovarian cancer has a poor outcome because it is usually detected at advanced tumor stages, and the majority of the patients develop disease relapse as a result of chemotherapy resistance. This most lethal gynecological malignancy metastasizes within the peritoneal fluid or ascites to pelvic and distal organs. In ovarian cancer progression and metastasis, small non-coding RNAs (ncRNAs), including long noncoding RNAs and microRNAs have been recognized as important regulators. Their dysregulation modulates gene expression and cellular signal pathways and can be detected in liquid biopsies. In this review, we provide an overview on circulating plasma and serum ncRNAs participating in tumor cell migration and invasion, and contributing to recurrence and metastasis of ovarian cancer. We will also discuss the development of potential, novel therapies using ncRNAs as target molecules or tumor markers for ovarian cancer.

## Introduction

The concept of a “RNA World” has yet to be completely established as a major step in the early stages of the evolution of living organisms, but it does form an intriguing step in the cell evolutionary pathway. Importantly, it involves the interaction of multiple forms of RNA cross-talk that will have been important in this era. However, how valid is this RNA concept? In the current situation, there are cells with a variety of RNAs, including messenger RNA (mRNA), transfer RNA (tRNA), ribosomal RNA (rRNA), small nuclear RNA (snRNA), small nucleolar RNA (snoRNA), small interfering RNA (siRNA), double strand RNA (dsRNA), RNA interference (RNAi), microRNA (miRNA), p-element induced Wimpy testis RNA (piRNA), circular RNA (circRNA), competing endogenous RNA (ceRNA) and long-non coding RNA (lncRNA), together with riboswitches^[1]^, ribozymes and RNA thermometers^[2]^ and with some cells open to attack by small RNA-containing viroids^[3]^. These are mainly plant pathogens, containing 246 to 467 nucleobases of highly complementary, circular, single-stranded, and non-coding RNAs without a protein coat^[4]^.

The majority of these RNAs fall into the category of non-coding RNAs (ncRNAs), i.e., they do not have an obvious protein-coding potential. More than 98% of the human genome has been considered to produce RNA transcripts that do not code for a protein^[5]^. The literature indicates that RNA-RNA crosstalk is alive and well, and forms an important aspect of healthy and diseased cell function, and especially in cancer^[6]^.

Many ncRNAs figure in the various stages of cancer initiation, progression and metastasis^[6-8]^. In particular, lncRNA, siRNA, miRNA, piRNA, and circRNA have been demonstrated to have roles in various aspects of ovarian cancer (OC) and its treatment. They are released into the blood circulation by apoptotic and necrotic cells^[9]^ and exist either cell-freely or associated with Argonaut proteins or they are actively secreted in exosomes into the blood^[10]^. The central role of exosomes has been highlighted by their ability to transfer miRNAs from cell to cell, to modulate the characteristics of the recipient cells, and induce tumor progression and spread^[11]^. Since mainly miRNA loci frequently map to fragile chromosomal regions harboring DNA amplifications, deletions or translocations, their expression is often deregulated during tumorigenesis^[12]^.

The topic on circulating plasma and serum ncRNAs will form the basis of the current review in which ncRNAs participating in tumor cell invasion and dissemination and contributing to metastasis of OC are described. Since no literature appears to exist on circulating siRNA, piRNA, and circRNA in the plasma and serum of OC patients, the focus of this review is on cell-free and exosomal lncRNA and miRNAs. In addition, consideration will be given primarily to the roles of ncRNAs in OC therapy.

## Ovarian cancer

### Features

OC is a deadly disease since the majority of patients are still diagnosed late, while early-stage disease is curable in nearly 90% of women, even in those with more aggressive histologic subtypes^[13]^. OC is the seventh most common cancer, the eighth most common cause of cancer death in women, and the second most common cause of gynecologic cancer death (after cancer of the cervix uteri)^[14]^. In 2018, worldwide, the number of new cases and deaths are estimated at 295,414 and 184,799, respectively (http://gco.iarc.fr/). Epithelial ovarian cancer (EOC) accounts for > 95% of OC, while non-epithelial cancers accounts for < 5% of OC including germ cell and sex-cord stromal cancer types, as well as rare, small cell carcinoma and ovarian sarcoma. EOC is histologically classified into 5 major subtypes: high-grade serous, low-grade serous, clear cell, endometrioid, and mucinous ovarian cancer^[15]^. Therapy decisions are based on disease stage, histology and biology, and comprise surgery in trying to avoid residual disease. Surgery permits accurate surgical staging, using the International Federation of Gynecology and Obstetrics (FIGO) staging system first published in 1973 and revised in 1988 and 2014^[16]^. Combined platinum-based chemotherapies are the preferred treatment strategies. Moreover, the administration of inhibitors against poly (ADP-ribose) polymerase molecules involved in the DNA damage-repair process has improved treatment regimens, particularly in recurrent patients^[17]^.

### Epithelial-mesenchymal transition

In OC, epithelial-mesenchymal transition (EMT) is associated with disease progression, chemoresistance and the acquisition of cancer stem cell properties. During EMT, epithelial cells go through phenotypic changes acquiring fibroblast-like mesenchymal features and cellular plasticity. They lose their cell polarity and adhesion to adjacent cells and the basement membrane, facilitating their migration through the extracellular matrix and settlement in other organs. This molecular reprogramming of the cell leads to the downregulation of epithelial-specific tight and adherents junction proteins like E-cadherin mediated by upregulation of the transcriptional repressors snail and slug, zinc finger E-box binding homeobox 1 (ZEB1) and ZEB2^[18]^ and Twist^[19]^. In conjunction with the loss of epithelial markers, new mesenchymal markers, like vimentin^[20]^ and N-cadherin^[21]^ are expressed^[22]^. EMT is induced by numerous signaling pathways, including TGF-β^[23]^, Wnt/β-catenin^[24]^, Notch^[25]^ and PI3K/Akt^[26]^. Moreover, hypoxia also influences the onset of EMT, mediated by the hypoxia-inducible factor-1 (HIF-1)^[27]^.

### Migration, invasion and peritoneal metastasis

**Figure 1:**
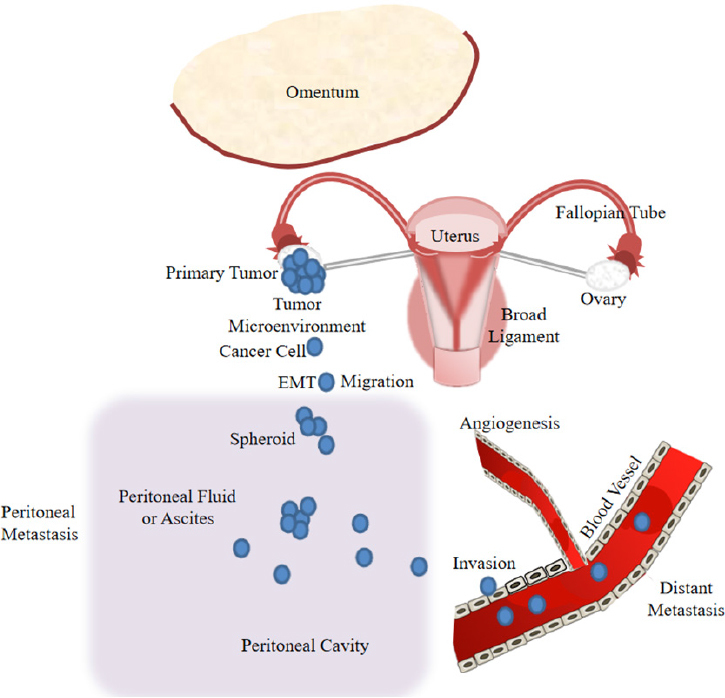
Model of ovarian cancer progression including cancer cell epithelial-mesenchymal transition (EMT), migration and invasion, and peritoneal and distant metastasis

Since the disease at early stages is usually asymptomatic, OC is usually diagnosed late when the cancer has already intra-peritoneally disseminated. OC invades either locally to adjacent tissues by direct extension from the primary tumor or to pelvic and distal organs within the peritoneal fluid or ascites as multicellular spheroids. Usually, most epithelial malignancies metastasize hematogenously and lymphogenously, however, metastasis of EOC occurs predominantly via transcoelomic dissemination^[28]^. Transcoelomic spread refers to OC metastasis into the peritoneal cavity. At first, metastasizing cells attach to the mesothelial monolayer outlining the peritoneal cavity, and subsequently, invade the submesothelial extracellular matrix of the underlying stroma. During peritoneal metastasis, increasing volumes of malignant ascites accumulate within the peritoneal cavity. The peritoneal metastasis leads to the omentum, a peritoneal fold that connects the stomach with abdominal organs, and the viscera [Figure 1]. The patients are typically treated by surgery and neoadjuvant chemotherapy consisting of a combination of a platinum drug and a taxane^[29]^.

### Lymph node and distant metastasis

EOC metastases frequently involve lymph nodes of which the abdominal lymph nodes are most frequently colonized. The FIGO ovarian cancer stage IIIC defines the metastatic involvement of retroperitoneal lymph nodes, while stage IVB defines the spread to inguinal lymph nodes, to the inside of the spleen or liver, and/or to other organs, such as the lungs and bones by metastases. However, distant metastases are seldom the main cause of death from metastatic EOC^[29]^.

## Chemotherapy and cancer stem cells

Usually, OC-related deaths are caused by transcoelomic dissemination of OC cells. OC invades to pelvic and distal organs within the peritoneal fluid or ascites forming multicellular spheroids [Figure 1]. Current first-line treatment for OC consists of complete debulking surgery followed by chemotherapy (carboplatin combined with paclitaxel)^[29]^. Carboplatin forms DNA cross-links and adducts^[30]^, while paclitaxel binds to the β-tubulin subunit of microtubules^[31]^. These activities of both agents induce cell damage and death. However, growing evidence indicates that these chemotherapeutic agents can also cause the enrichment of cancer stem cells. Hence, cancer stem cells may be able to survive chemotherapy and demonstrate increased chemoresistance by modifying various stem cell signaling pathways, such as Wnt and Notch, to then, drive tumors to relapse and metastasize^[32]^. These resistant features of stem cells are attributed to high expression levels of drug efflux pumps, such as ATP-binding cassette transporters, high DNA repair capacities and high levels of anti-apoptotic proteins^[33]^. OC stem cells play a central role in the formation of multicellular spheroids during the transcoelomic peritoneal dissemination of OC. In the malignant ascites, spheroids were shown to be enriched in cancer stem cells that are predominantly responsible for OC transcoelomic metastasis^[34]^. Further investigations on the biology of OC stem cells in association with cancer metastasis and resistance to chemotherapy are needed to develop effective therapeutic modalities, such as new targeted therapies that use ncRNAs as prognostic markers or target molecules and avoid activation of cancer stem cells.

## Serum tumor markers

### CA125

Cancer antigen 125 (CA125) encoded by the mucin gene MUC16 is a transmembrane glycoprotein produced by the coelomic epithelium. CA125 is cleaved outside the cell membrane and then shed into body fluids where it can be detected with double determinant immunoassays^[35]^. Measurements of this serum marker are routinely used in clinical practice. Aberrant serum levels of CA125 have been detected in endometriosis^[36]^ and pelvic inflammatory disease^[37]^. In particular, they are the standard of care in performing OC diagnosis, and to assess response and relapse to treatment. However, CA125 assessment shows low sensitivity and specificity to diagnose EOC at early stages of disease. CA125 is only elevated in approximately 50% of stage I, and 70%-90% of advanced diseases. Notably, its sensitivity increases during cancer progression^[17]^. In general, response to treatment is associated with a decrease in serum CA125 values by half, whereas a doubling of CA125 indicates drug resistance and disease progression. Persistent increase in CA125 values in patients after primary chemotherapy predicts persistence of residual disease with 90% accuracy. Persistently rising CA125 is a highly specific indicator of recurrent disease^[38]^. A systematic review of the literature identifying 21 relevant original articles to interpret serial CA125 concentrations reported that the median sensitivity of the investigated criteria for recurrence was 57% during primary therapy and 85% during follow-up. The performances of the CA125 assessment criteria showed low sensitivities and low ability to exclude tumor growth. The chance of developing clinical progression following CA125 progression was however high (predictive value 90%-100%)^[39]^.

### HE4

The human epididymis protein-4 (HE4) encoded by the WAP four-disulfide core domain 2 gene is also a glycoprotein and commonly used as a tumor marker in OC. Its plasma levels are increased in gynecological pathologies and are useful in prognosis as well as in assessing the degree of differentiation and clinical progression of OC^[40]^. Dewan *et al*.^[41]^ compared the sensitivity and specificity of H4 values with those of CA125 using serum samples from OC patients and patients with benign ovarian diseases. To predict OC, a receiver operating characteristic curve (ROC) was used to calculate a cut-off value of serum HE4 and CA125 values. Their measurements revealed that HE4 values had a similar sensitivity (83.6%) to CA-125 (85.10%), but HE4 (100%) had a higher specificity than CA125 (90.48%). The combination of serum HE4 and CA125 values improved the sensitivity to detect ovarian cancer up to 92.54%. The sensitivity of HE4 (92.61%) was superior to CA125 (63.41%) to detect early stage ovarian cancer^[41]^. Thus, measuring the serum values of HE4 along with CA125 may provide improved accuracy for OC detection, mostly in the early stages.

## ncRNAs involved in ovarian cancer

### miRNAs and mRNAs

A miRNA is a small non-coding RNA molecule about 22 nucleotides long and functioning in both RNA silencing and post-transcriptional regulation of gene expression^[42]^. In doing so, it downregulates the expression of protein-encoding genes as well as genes encoding for lncRNAs. This RNA is incorporated into an argonaute-containing protein complex termed RISC (RNA-induced silencing complex) that is involved in mRNA silencing as a consequence of the miRNA base-pairing with complementary sequences within mRNA molecules. This leads to either cleavage of the mRNA strand into two pieces, or destabilization of the mRNA through shortening of its poly(A) tail, or less efficient translation of the mRNA into proteins by ribosomes^[43]^. Thus, part of the RNA cross-talk can occur through interaction of the mRNAs and miRNAs of which there are 1,917 listed for *Homo sapiens* (www.miRBase.org) though Griffiths-Jones *et al*.^[44,45]^ have listed 2588 annotated miRNAs in the human genome.

### lncRNAs

Transcripts with lengths exceeding 200 nucleotides that are not translated into protein are called lncRNAs^[46]^. There are large numbers of lncRNAs present with >210,000 having been recorded by Ren *et al*.^[47]^ of which 106,063 are human related (LncRNAWiki, 2015) though only 16,840 were recorded in Gencode (https://www.gencodegenes.org). There is a debate as to whether or not lncRNAs do encode proteins with some authors who indicate that they do code for proteins^[48-51]^. It is also possible that the products of lncRNAs are either unstable or have no biological function - or both^[52]^. Only 1,867 human lncRNAs have been experimentally demonstrated to be biologically active in humans^[53]^.

The roles of lncRNAs are complex and include imprinting, X-chromosome inactivation, the organization of the nuclear architecture, gene transcription and epigenetic chromatin modification^[6,54,55]^.

Studies on OC using deep RNA sequencing have revealed 53 lncRNAs associated with OC of which 27 were upregulated and 26 downregulated, but of which only 25 could be directly related to a clinical application^[56]^. It is clear, even from these few lncRNAs that there is a need to relate them more specifically to a particular stage in the development and treatment of OC in order to determine their actual roles in the processes of tumor initiation, cell migration and metastasis.

### piRNAs

The p-element induced Wimpy testis RNAs (piRNA) have been recently found to be involved in a number of aspects of cell biology and cancer. The human genome appears to contain 23,439 of these ~26-30 nucleotide long molecules^[8]^, i.e., closer to that of the protein coding mRNAs, but greater than the 2,588 listed miRNAs^[57-61]^. In addition to their roles listed in Table 1, piRNAs have been demonstrated to be involved in cancer, including OC. Four PIWI proteins have been identified, namely PIWIL1/HIWI, PIWIL2/HILI PIWIL3/HIWI3 and PIWIL4/HIWI2 that have been applied in OC diagnosis and prognosis^[62]^. Moreover, they have been variously used for diagnosis, prognosis, migration and invasion in a variety of cancers (reviewed in^[8]^). Nevertheless, in all cancer types, the presence of these PIWI proteins correlates with aggressive cancers and poor clinical outcomes, including OC^[62]^. Li *et al*.^[72]^ and Yao *et al*.^[73]^ have examined the role of piR-651 in non-small cell lung carcinoma (NSCLC). Its upregulation was associated with cancer progression in NSCLC patients whilst its overexpression in A549 lung cancer cell line increased both tumor growth and metastasis. Given that overexpression of piR-651 also results in the increase of cyclin D1 and CDK4, and hence a likely involvement in the cell cycle arrest, the authors suggest that piR-651 could act as a possible oncogene in NSCLC. Moreover, the results of Law *et al*.^[74]^ imply that piR-Hep1 acts as an oncogene in liver cancer by promoting cell proliferation, migration and invasion.

**Table 1:** Possible roles of piRNAs

piRNAs	Functions	Ref.
ncRNAs bind to PIWI subfamily Argonaute proteins	Epigenetic changes in germ cell lines	[61,63-66]
piRNA/PIWI complex	Transposon silencing heterochromatin modification	[67,68]
piRNAs	DNA methylation of transposons and specific genes	[69,70]
	Degradation of retrotransposon- linked mRNA	[71]

piRNA: p-element induced Wimpy testis RNA; mRNA: messenger RNA; ncRNAs: non-coding RNAs

Using OC cell lines and its resistant cell lines, Wang *et al*.^[75]^ demonstrated that stem cell protein Piwil2 was also enhanced in the two completely different cisplatin-resistant cell lines. The enhanced sensitivity to cisplatin and decreased efficiency for removing cisplatin-induced DNA intra-strand crosslinks in OC cells were induced by Piwi12 knock-down. The authors suggest that the cellular cisplatin resistance could be due to overexpression of Piwil2 in some cancers. Furthermore, the enhanced chromatin condensation could also affect normal DNA repair.

### ceRNAs

The degree of crosstalk between miRNA/mRNA has been extensively discussed by Salmena *et al*.^[76]^ who proposed a competing endogenous RNA (ceRNA) hypothesis in which a change in the expression levels of one ceRNA will modify the amount of its miRNA regulator resulting in changes to the expression levels of other target ceRNAs of the initial miRNAs.

A ceRNA involves immediate interactions between RNA molecules due to interactions with common miRNAs^[77-79]^. CeRNAs include protein coding RNAs (mRNAs), as well as lncRNAs^[80,81]^, pseudogenes^[82,83]^ and circRNAs (see below). Usually, when linked to Argonaute proteins in the RNA-induced silencing complexes, miRNAs can modify protein synthesis by binding to miRNA response elements (MREs) that are present on the 3’UTR of mRNAs leading to either mRNA degradation or inhibition of the mRNA translation into proteins^[42]^. It appears that mRNA normally contains a number of MREs so leading to the situation where a single mRNA can be targeted by a number of miRNAs - and vice versa^[84]^. The outcome is a variety of degrees of protein synthesis control as opposed to the simpler idea of a miRNA just blocking a mRNA activity. As well as being important components of the control of protein synthesis in healthy cells^[85]^, ceRNAs appear to play an important role in tumor cells and tissues^[86-88]^ with modification of miRNA concentrations possibly resulting in dysregulation of either oncogenes and/or of tumor suppressors^[89]^. ceRNA involvement in cancer has been well-reviewed by a number of scientists, e.g., Karreth & Pandolfi^[90]^, Tay *et al*.^[91]^, Qi *et al*.^[87]^, Chan and Tay^[6]^.

A number of computational systems have been devised to determine ceRNA involvement in cancer in which miRNAs are considered to be the only type of gene regulators. However, Do and Bozdag^[92]^ have suggested that such miRNAs should be considered together with other important types of regulators, e.g., DNA methylation, transcription factors and copy number variation. As a result, these factors have been included in a new computational pipeline model: Cancer-associated ceRNAs interaction networks, Cancerin in which both lncRNAs and mRNAs are considered as ceRNAs. An easy-to-use R software for Cancerin is freely available at https://github.com/bozdaglab/Cancerin^[92]^. To determine potential ceRNAs, Cancerin was employed with data from both miRNA interactions and their profiles. Such an approach was applied using data from breast invasive carcinoma, kidney renal clear cell carcinoma and head and neck squamous cell carcinoma. The data obtained indicated that ceRNAs were enriched with cancer-related genes. Moreover, the behavior of clusters of related RNAs yielded data implying the presence of ceRNA networks related to cancer-associated biological activities^[92]^.

Thus, the recent ceRNA studies invoke a further degree of networking between RNAs in both healthy and tumor cells.

### CircRNAs

Circular RNAs (circRNAs) were first demonstrated in 1976 by Sanger *et al*.^[4]^ who showed the potato spindle tuber RNA virus - a viroid - to be circular. Subsequently, circRNAs were identified in yeast mitochondria by Arnberg *et al*.^[93]^. Since then, circRNAs have been shown to be present in the nuclei and cytoplasm of a variety of species and cell types, especially in human healthy and cancer cells, so becoming an important member of the ncRNA:RNA regulatory networks^[6,94,95]^. CircRNAs are formed in the nucleus and yield three different types that are derived from either introns or exons or a combination of one intron-derived fragment and one exon-derived fraction linked together^[96]^. In addition, some circRNAs can act as pseudogenes. In a sense, their origins define their functions in that they are able to act as functional mRNAs leading to the production of functional proteins^[97-100]^. Intron-derived circRNAs tend to remain in the nucleus where they have been demonstrated to regulate RNA transcription through either binding to RNA polymerase II^[101]^ or by attaching to the cognate DNA^[102]^. The pseudogenes can be reverse transcribed to form cDNA that can be integrated into the genome. The majority of circRNAs are found in the cytoplasm where they function as miRNA “sponges” resulting in miRNA expression inhibition^[103]^. Equally, they can act as reservoirs of miRNA that can be released so stabilizing specific miRNA levels in, e.g., tumor cells^[96]^. Furthermore, Ahmed *et al*.^[104]^ demonstrated 67,580 circRNA isoforms in FIGO stage IIIC ovarian cancer with more circRNAs being differentially expressed between the primary OC and the metastatic OC than was seen with mRNAs. It was suggested that over-expression of such circRNAs could result in a regulation of downstream target genes due to the circRNAs behaving as carcinogenetic miRNA sponges. However, the opposite was determined in a renal cell carcinoma study where a circRNA (circHIAT1) acted as a miRNA reservoir, stabilizing miRNA expression^[96]^. Thus, together with mRNA and miRNA, circRNA forms an important crosstalk network that regulates cellular events including cell division, cell mobility and metastasis and may be considered to be a member of the ceRNAs (see above).

Although there are limited studies of circRNAs, they appear to have important roles in cancer. Xu^[95]^ has listed a number of circRNAs for a variety of cancer types and in particular, OC^[100]^ that are either up- or down-regulated and appear to have an impact on signalling pathways as well as to act as sponges and reservoirs. In this regard, Ahmed *et al*.^[104]^ have noted that cirs-7 acts as a sponge in OC where it appears to competitively inhibit linear splicing and sponge function of miRNAs and modulate the expression of metastasis inducing genes, contributing to OC metastasis.

Cancer-related chromosomal translocation fusion circRNAs (f-circRNA) have also been identified. Following chromosomal translocation of, e.g., gene A and gene B, the fusion and back-splice junctions of a gene A exon fuses with a gene B exon to yield a f-circRNA^[100,105]^.

### SiRNAs and mRNAs

Small/short/silencing RNAs (siRNAs) are of interest for two reasons. The first is that they are concerned with gene expression modifications involved with the initiation and maintenance of cancer together with roles in cell motility and metastasis. The second is the possible exploitation of modified siRNA in the control of cancer cell development and metastasis. They were initially discovered in Caenorhabditis elegans^[106]^ and plants^[107]^.

They are 20-25 base pair, double stranded RNA molecules that operate via the RNA interference pathway preventing the translation of mRNA derived from genes with complementary nucleotide sequences through its degradation after transcription. Hence, they resemble very much miRNAs. Moreover, they are formed by the enzyme Dicer from long dsRNA that can be derived from hairpin RNA. Once in the cytoplasm, they link to the RISC. On binding, the strand that is less thermodynamically stable due to base paring at the 5’ end stays in the RISC complex and becomes the strand that will bind to the target mRNA that is to be cut. Major differences to miRNAs are the total complementarity with and high target specificity of siRNA, and the cleaving of mRNA prior to translation^[108]^.

SiRNA provides a vehicle that can be either modified or synthesised *de novo* for exploitation *in vivo* to alter the levels of specific gene activities linked to cancer. Examples from OC are the targeting of human epidermal growth factor receptor 2 (HER-2)^[109]^ and EphA2^[110]^ receptors. However, there are difficulties with this approach due to the need for the siRNA to bind to RISC. A mechanism to ensure that this will occur involves the siRNA sequence being altered so that a short loop between the two strands is introduced to yield a short hairpin RNA (shRNA). This can then be modified into a functional siRNA through the normal activity of Dicer. Successful delivery also requires a vehicle to avoid non-specific effects and, e.g., it being treated as a viral product and so inducing an immune response. Current delivery methods^[111]^ include either polymer-mediated^[112-114]^ or peptide-based^[115-117]^ or lipid-based^[118-120]^ or siRNA conjugate delivery systems^[121-125]^. The application of siRNAs in the attempt to interfere with tumors has been primarily at the cell culture level rather than the whole organism^[108]^. Nevertheless, this approach does open up a new way to modify tumor cells and a broad variety of delivery systems have been employed in studies on OC^[126-133]^.

## Circulating ncRNAs involved in advanced ovarian cancer

Table 2 gives an overview on the deregulated circulating miRNAs detected in plasma, serum, ascites, urine and exosomes from OC patients and their function in EMT, migration, invasion and metastasis.

**Table 2:** Circulating miRNAs and lncRNAs in advanced ovarian cancer

miRNAs	Source	Regulation	Association with	Ref.
miR-7	Serum	Up	Metastasis	[134]
miR-30a-5p	Urine	Up	Lymph node metastasis	[135]
miR-30a-5p	Urine exosomes	Up	Migration	[135]
miR-92	Serum	Up	Lymph node metastases	[136]
miR-99a-5p	Serum exosomes	Up	Invasion	[137]
miR-101	Serum exosomes	Down	Migration	[138]
miR-125b	Serum	Up	Metastasis	[139]
miR-130a	Serum	Up	Recurrence	[140]
miR-145	Serum	Down	Migration	[141]
miR-145	Serum	Down	Metastasis	[142]
miR-148a	Plasma	Down	Lymph node metastases	[143]
miR-183	Serum	Up	Lymph node metastases	[144]
miR-193a-5p	Serum	Down	Lymph node metastases	[145]
miR-193a-5p	Plasma	Down	Recurrence	[146]
miR-199a	Serum	Down	Metastasis	[147]
miR200a, miR200b, miR200c, miR141, miR429, miR203a, miR34b, miR34a	Plasma	Up	EMT	[148]
miR-200b, miR-200c	Serum exosomes	Up	Lymph node metastasis	[149]
miR-200b, miR-200c	Serum	Up	Lymph node metastasis	[150]
miR-375	Serum exosomes	Up	Lymph node metastases	[151]
miR-376a	Serum	Up	Metastasis	[152]
miR-423-5p	Plasma	Down	Invasion	[153]
miR-429	Serum	Up	Migration, invasion	[134]
miR-590-3p	Plasma	Up	Metastasis	[154]
miR-940	Ascites exosomes	Up	Migration	[155]
miR-1273g-3p	Serum	Down	Recurrence	[156]
lncRNAs				
MALAT1	Serum exosomes	Up	Metastasis	[157]
LINK-A	Plasma	Up	Metastasis	[158]
LINK-A	Serum	Up	Metastasis	[159]

miRNAs: microRNAs; lncRNAs: long-non coding RNAs; EMT: epithelial-mesenchymal transition; MALAT1: metastasis-associated lung adenocarcinoma transcript 1

### Circulating miRNAs in ovarian cancer recurrence

Despite the improvement in treatment and overall survival, a fraction of EOC patients at advanced tumor stages fails to respond to primary therapy and relapses occur in 70% of cases^[160]^. Several miRNAs associated with OC recurrence were identified by Wang *et al*.^[161]^, and included miR-375, miR-141 and miR-27b.

Concerning the matter of circulating miRNAs, Chao *et al*.^[140]^ analyzed candidate miRNAs in the serum of patients with clear cell OC using quantitative PCR (qPCR) for 270 miRNAs. They identified elevated serum levels of miR-130a in early disease recurrence before the serum marker CA125 was found to be elevated^[140]^. In high-grade serous OC, miR-130a is trans-activated by NF-κB and targets the tuberous sclerosis gene TSC1 resulting in the upregulation of the mammalian target of rapamycin (mTOR) signaling pathway which is associated with poor prognosis. In this context, miR-130a drives proliferation, cell invasion and metastasis of OC cells^[162]^. The deregulation of miR-130a is also involved in the development and regulation of cisplatin resistance. In these resistant OC cells, it regulates the multidrug resistance 1 (*MDR1*) and phosphatase and tensin homolog (*PTEN*) gene expression^[163]^.

In addition, Günel *et al*.^[156]^ dealt with the association of circulating miRNAs with OC recurrence. Using microarray and real-time PCR, they compared miRNA expression in serum samples of recurrent EOC patients with that of healthy women. The serum levels of miR-1273g-3p were significantly lower in recurrent EOC patients than in healthy women, and could differ between the two cohorts with an area under the ROC curve (AUC) of 0.7. Pathway analysis showed that miR-1273g-3p is involved in several cell processes including apoptosis, cell proliferation, migration and cell invasion. Downregulation of miR-1273g-3p led to the high gene expression of matrix metalloproteinases (*MMP*-2 and *MMP*-9), tumor necrosis factor alpha (*TNF*-*α*), collagen type I alpha-1 (*CO1A1*) in OC^[156]^. These gene products are known to regulate OC progression. MMPs degrade a variety of proteins in the extracellular matrix and consequently, contribute to cell invasion and metastasis of OC^[164]^. Overexpression of *TNF*-*α* is associated with the increasing tumor grade^[165]^, while epigenetic silencing of *COL1A1* is linked to the development of platinium-based resistance in OC^[166]^.

### Circulating miRNAs in epithelial-mesenchymal transition, cell exfoliation, migration and invasion

The signaling pathway P13K (phosphoinositide-3 kinase)/AKT/mTOR is considered to have a crucial role in EMT, migration and invasion in OC, and is abnormally activated in different cancer types^[167]^. Many miRNAs, including miR-130a, miR-497 and miR-199 have been found to influence this pathway^[168]^.

Using Northern blot and real-time PCR, Wu *et al*.^[141]^ quantified circulating serum miR-145, and indicated that this miRNA is downregulated in serum samples as well as in OC tissues and cell lines, compared to the corresponding healthy sources. Functional studies suggested that miR-145 overexpression inhibited cell growth and invasion, and induced cell apoptosis of OC. MiR-145 was found to negatively regulate ribosomal protein S6 kinase, polypeptide 1 (P70S6K1) involved in the mTOR pathway and the oncogene MUC1 by directly targeting their 3’UTRs^[141]^. Moreover, downregulation of miR-145 regulates cell invasion as well as angiogenesis promoting OC metastases^[169]^. Tang *et al*.^[153]^ showed that miR-423-5p also inhibited the invasion of OC and was downregulated in plasma as well as in tissue of OC patients, compared with healthy individuals. Ectopic overexpression of miR-423-5p reduced proliferation and invasion of OC^[153]^. MiR-423-5p was reported to be a sponge for the lncRNA LINC00319 that promotes proliferation, migration and invasion of OC cells. Furthermore, miR-423-5p directly targeted nucleus accumbens associated 1 (NACC1). Real-time PCR and Western blot results demonstrated that LINC00319 upregulated NACC1 expression through inhibiting miR-423-5p in OC cells^[170]^. Accordingly, both miR-145 and miR-423-5p may serve as a diagnostic indicator for cell invasion and functions as a tumor suppressor in OC.

Márton *et al*.^[148]^ screened miR-200a, miR-200b, miR-200c, miR-141, miR-429, miR-203a, miR-34b and miR-34a which are involved in EMT, and found significantly higher plasma levels of these miRNAs in OC patients than in patients with benign ovarian tumors and disease-free healthy volunteers.

In the blood circulation, miRNAs do not only exist as cell-free molecules, they also circulate in exosomes. Using miRNA microarray and real-time PCR, Yoshimura *et al*.^[137]^ demonsrated that the serum levels of exosomal miR-99a-5p were elevated in EOC patients compared with patients with benign tumors and healthy women. ROC analysis showed a sensitivity and specificity of 0.85 and 0.75 (AUC of 0.88), respectively, with a cut-off of 1.41 for detecting EOC. Treatment with EOC-derived exosomes significantly increased miR-99a-5p expression in human peritoneal mesothelial cells (HPMCs). HPMCs transfected with miR-99a-5p promoted OC invasion and exhibited increased expression levels of fibronectin and vitronectin. Xu *et al*.^[138]^ found that the levels of miR-101 were altered in serum exosomes as well as in tissue samples. Using dual-luciferase assay and immunoblotting, they showed that miR-101 could repress the expression of brain-derived neurotrophic factor (BDNF) by targeting its 3’-UTR. By transwell assays, they indicated that the reduction of miR-101 was mostly related to significantly enhanced OC cell migration, suggesting that miR-101 may repress cell migration via binding to BDNF^[138]^. BDNF and its receptor tropomyosin receptor kinase B (TrkB) are usually upregulated in diverse cancer types, and stimulate a series of downstream pathways resulting increasing cancer cell growth, proliferation, survival, migration and EMT^[171]^. Moreover, miR-101 inhibited EMT and invasion of OC cells by down-regulating expression of the E-cadherin repressors ZEB1/ZEB2 and suppressor of cytokine signaling-2 (SOCS-2), respectively^[172,173]^.

OC often manifests itself by excessive fluid accumulation, namely ascites in the peritoneal cavity. Ascites-derived miRNAs, such as miR-200a, miR-200c, miR-141, miR-429 and miR-1290, may be related to OC progression^[174]^. Besides, ascites from advanced-stage and serous OC contain large numbers of tumor-derived microparticles, such as exosomes, that promote cell migration^[175]^. Chen *et al*.^[155]^ observed high levels of miR-940 in exosomes derived from ascites of EOC patients. Hypoxia, a common feature of solid tumors, induced the expression of exosomal miR-940. When delivered by exosomes to macrophages, overexpression of miR-940 stimulated M2 phenotype polarization which promoted EOC proliferation and migration.

### Circulating miRNAs in metastases

A set of specific genes and signaling pathways that are under control of miRNAs regulate metastasis. For example, miR-145, miR-31, miR-506, miR-101, miR-200, miR-214 and miR-25 are most essential for metastasis of OC and are discussed in detail in the review by Braga *et al*.^[176]^.

MIR-92 was reported to contribute to angiogenesis, since it stimulates the angiogenic factor vascular endothelial growth factor by inhibiting the von Hippel-Lindau gene product (VHL) in EOC. Guo *et al*.^[136]^ detected higher serum levels of miR-92 in EOC patients than in healthy women and a significant association of miR-92 expression with regional lymph node metastasis.

Using real-time PCR, Gong *et al*.^[143]^ detected lower plasma levels of miR-148a in OC patients compared with healthy controls. In particular, low plasma yields were associated with lymph node metastasis, whereas patients with a high level of miR-148a had a longer survival time than those with a low level. Moreover, cell experiments confirmed that miR-148a could inhibit OC proliferation, migration and invasion. The significant association of serum miR-183 with lymph node metastases in OC was documented by Chen *et al*.^[144]^. High serum levels of miR-183 were also associated with poor overall survival in EOC patients^[144]^. MiR-183 may exert its tumor-promoting effects on OC among others by regulating the transcription factor Smad 4 via the TGF-β/Smad4 signaling pathway^[177]^.

A diagnostic model of serum miR-193a-5p and the tumor markers CA125 and HE4 for EOC was evaluated by Ren *et al*.^[145]^ that significantly correlated with lymph node metastasis. The AUC of the risk model for distinguishing EOC patients from healthy individuals was 0.996 which was higher than any single biomarker^[145]^. Apart from miR-193a-5p, the deregulated plasma levels of which were also reported in recurrent platinum resistant OC patients^[146]^, the improvement of the diagnostic power of CA125 and HE4 for OC was also shown to be mediated by serum exosomal miR-375 and miR-1307. Su *et al*.^[151]^ detected that both miR-375 and miR-1307 were significantly up-regulated in serum exosomes of OC compared with ovarian benign and healthy groups. Notably, exosomal miR-375 correlated with lymph node metastasis of OC^[151]^. In this regard, miR-375 was reported to target yes-associated protein 1 (YAP1) and interact with lncRNA MLK7-AS1 resulting in modulating EMT and promoting the expression of the transcriptional repressor Slug, a key player in EMT^[178]^.

As shown by Yang *et al*.^[152]^, the serum levels of miR-376a were significantly higher in OC patients and associated with the clinical stages of OC. Overexpression of miR-376a stimulated the proliferation, migration and invasion of OC cells, while inhibition of miR-376a expression blocked these processes. Western blotting and luciferase assays revealed that miR-376a targets the transcription factor Krüppel-like factor 15 (KLF15) and caspase-8. Data from nude mice further demonstrated the stimulatory role of miR-376a in OC progression and metastasis^[152]^. Our laboratory^[134]^ supports these findings, and found that the serum levels of miR-376a correlated with peritoneal metastasis outside the pelvis and distant metastasis. In the same study, we also showed that the deregulated serum levels of miR-25, miR-93, miR-7 and miR-429 discriminated EOC patients from healthy women with a sensitivity of 93% and a specificity of 92%. Overexpression of miR-429 that targets ZEB1^[179]^ led to suppression of migration and invasion, whereas overexpression of miR-7 resulted in increased migration and invasion in OC cells. Increased serum levels of miR-7 were associated with lymph node, peritoneal and distant metastases^[134]^. Our laboratory^[149,180]^ also investigated the serum levels of exosomal miR-373, miR-200a, miR-200b and miR-200c. We detected that the levels of miR-200b and miR-200c were higher in patients with FIGO stage III-IV including lymph node metastasis than FIGO stages I-II. The increased levels of miR-200b and miR-200c were also associated with CA125 values and a shorter overall survival^[149,180]^. In line with our findings are the data by Zuberi *et al*.^[150]^. They showed that miR-200b and miR-200c are associated with aggressive tumor progression, and reliable markers to predict the prognosis and survival in EOC patients. They observed elevated levels of cell-free miR-200b and miR-200c in the serum of EOC patients in comparison with matched normal controls. Moreover, patients with lymph node metastasis displayed a significant elevation of miR-200c^[150]^. To date, several studies have publicized that an increased expression of miR-200c is related to a decreased overall survival. This occurs presumably through the upregulation of EMT^[181]^. In contrast, Hurteau *et al*.^[182]^ exposed that overexpression of miR-200c led to a reduced expression of ZEB1 and thus, to an increased expression of E-cadherin, inhibiting EMT.

Zuberi *et al*.^[139]^ investigated several miRNAs, among others miR-125b^[139]^, miR-145^[142]^ and miR-199a^[147]^ in the serum of EOC patients. They detected that the expression of miR-125b was significantly upregulated whereas expression levels of miR-145 and miR-199a were downregulated in EOC patients in comparison with healthy women. All three serum miRNAs correlated with lymph node and distant metastasis^[139,142,147]^. MiR-125 does not only play a role in metastasis, but its up-regulation also causes a marked inhibition of cisplatin-induced cytotoxicity and apoptosis, namely, a subsequent increase in the resistance to cisplatin in OC cells. The pro-apoptotic Bcl-2 antagonist killer 1 (Bak1) is a direct target of miR-125b and down-regulation of Bak1 suppressed cisplatin-induced apoptosis and led to an increased resistance to cisplatin^[183]^. Several target mRNAs are known for miR-145. For example, miR-145 inhibited metastasis by targeting the cell adhesion molecule metadherin (MTDH)^[184]^ and sensitized OC cells to paclitaxel by targeting the transcription factor Sp1 and cyclin dependent kinase 6 (Cdk6)^[185]^. Surprisingly, the downregulation of miR-145 also played a tumor suppressive role. In this context, Hu *et al*.^[186]^ demonstrated that circ-TCH acted as a ceRNA (competing endogenous RNA) to sponge miR-145, increased the level of the RasGTPase RASA1, and inhibited the malignant progression of OC cells via the circ-ITCH-miR-145-RASA1 axis *in vitro* and *in vivo*^[186]^. Besides, the target mRNA of miR-199a is TGF-β2. Song *et al*.^[187]^ demonstrated that emodin, a natural anthraquinone directly activated the expression of the transcription factor forkhead box D3 (FOXD3) and sequentially activated miR-199a, which in turn suppressed the expression of TGF-β2 to reduce cell viability and colony formation of OC cells.

In the study by Salem *et al*.^[154]^, expression and function of miR-590-3p were determined in EOC. The plasma levels of miR-590-3p were significantly higher in EOC patients than in subjects with benign gynecologic disorders. Transfection of miR-590-3p increased cell proliferation, migration and invasion. *In vivo* studies revealed that miR-590-3p accelerated metastasis. Using a cDNA microarray, this laboratory identified forkhead box protein A2 (FOXA2) and the extracellular matrix component versican (VCAN) as top genes downregulated and upregulated by miR-590-3p, respectively^[154]^.

In addition to plasma, serum and ascites, urine is a source for miRNA measurements. However, urine is rarely used for ncRNA quantification because of its shortcomings. To reach the bladder, miRNAs have to pass through the renal filtration system. The kidney barrier has been shown to be permeable for molecules, but only complexes smaller than 6.4 nm in diameter and with a molecular weight ≤ 70 kDa corresponding to molecules of about 100 bp in size can pass through it and enter the nephron^[188]^. To determine the clinical value of urinary miRNAs for serous OC, Zhou *et al*.^[135]^ performed a miRNA microarray and showed that only miR-30a-5p was upregulated whereas 37 miRNAs were downregulated in the urine samples of OC patients when compared to healthy controls. The upregulation of urinary miR-30a-5p was associated with lymphatic metastasis and significantly reduced after surgical removal of the serous adenocarcinoma, indicating that urinary miR-30a-5p could be derived from the OC tissue. Amazingly, miR-30a-5p was also detected in exosomes from the urine of serous OC patients so supporting the idesa of a pathway for excretion into the urine. Possibly, the deformability of exosomes may allow their passage through the kidney barrier. Finally, *in vitro* analysis also showed that the knockdown of miR-30a-5p significantly inhibited the proliferation and migration of OC cells^[135]^.

### Circulating lncRNAs in metastasis

In their review, Zhan *et al*.^[56]^ provided a good overview on the dysregulated expression of lncRNAs and their potential clinical implications in OC. Among others, they outlined the association of HOX transcript antisense intergenic RNA (HOTAIR) and an antisense noncoding RNA named ANRIL with lymph node metastasis. So far as we know, there are only 3 publications on circulating lncRNAs in OC patients.

Metastasis-associated lung adenocarcinoma transcript 1 (MALAT1) is a well-known lncRNA associated with cancer angiogenesis and metastasis^[189]^. In EOC patients, Qiu *et al*.^[157]^ found elevated serum levels of exosomal MALAT1 that highly correlated with an advanced and metastatic phenotype of EOC and were an independent predictive factor for EOC overall survival. In addition, their data provided evidence that EOC cells transfer MALAT1 via exosomes to recipient endothelial cells and influence these cells by stimulating angiogenesis-related gene expression^[157]^.

In their study, Ma *et al*.^[158]^ investigated the relationship between long intergenic non-protein coding RNA (LINK-A) and TGF-β1. They found that LINK-A and TGF-β1 were up-regulated in the plasma of OC patients compared with healthy women. Plasma levels of LINK-A were positively associated with plasma TGF-β1 in OC patients, but not in healthy controls and correlated with distant tumor metastasis. LINK-A overexpression promoted cell migration and invasion by up-regulating TGF-β1 and activating the TGF-β pathway in OC cells^[158]^. Finally, Zhang *et al*.^[159]^ also found that the serum levels of LINK-A were higher in OC patients than in healthy controls and associated with metastasis. LINK-A overexpression promoted migration and invasion, and upregulated the expression of HIF1a in OC cells^[159]^.

### Potential therapies using ncRNAs

MiRNAs have been suggested as biomarkers and therapeutic agents for OC. The development of miRNA-based therapies aims at regulating the aberrant expression of miRNAs in cancer. In this regard, modulation of miRNA expression and their signaling pathways may be used to potentiate treatment regimens in OC. Since the downregulation of tumor-suppressive miRNAs and the upregulation of oncogenic miRNAs contribute to cancer development, progression and metastasis, restoring the miRNA tumor-suppressive function and inhibiting the oncogenic function may be used as a novel strategy in cancer treatment. MiRNA-based therapies can apply either synthetic miRNA-like molecules, including miRNA mimics and miRNAs encoded in expression vectors, which restore miRNA expression or anti-miRNAs known as antagomirs including antisense oligonucleotides, miRNA sponges and anti-miRNA peptides which inhibit miRNA expression. As delivery systems of miRNAs, in particular, polymer-based vesicles have been widely investigated because of their easy synthesis and flexible properties^[190]^.

MiR-34 is an example for restoring the miRNA tumor-suppressive function. This miRNA is significantly downregulated in numerous different cancer types including OC and regulates multiple oncogenic pathways^[191]^. A first-in-human phase I clinical trial (NCT01829971) was conducted to investigate the safety, pharmacokinetics and clinical activity of a liposomal formulation of miR-34 mimic (known as MRX34)^[192]^. In a subset of patients with refractory advanced solid tumors MRX34 was well tolerated and displayed antitumor activity. However, in September 2016, the sponsoring company (Mirna Therapeutic, Inc.) stopped the enrollment and dosing of MRX34 in the clinical study following multiple immune-related severe adverse effects in patients dosed with MRX34^[193]^. This initial clinical experience with miR-34 replacement therapy is of course discouraging, but the investigations of miRNAs as therapeutic approach for the treatment of OC is still a promising research field.

The development of successful miRNA-based gene therapies has some challenges, such as the rapid degradation of miRNAs or anti-miRNAs by cellular nucleases and the poor cellular uptake. Since miRNAs only require partial sequence complementarity to their target mRNA, non-specific binding of bystander mRNAs may lead to unspecific effects, toxicity and/or unfavorable immune response. Furthermore, the administration of antagomirs may adversely affect physiological functions normally regulated by these miRNAs.

Although there are blood-based protein markers, such as CA125 and HE4, their specificity and sensitivity for OC are restricted. Since blood constitutes a pool of circulating miRNAs derived from different sources, e.g., from heterogeneous areas of the primary tumor, metastases or other organs affected by tumor burden, their minimally invasive quantification is qualified for keeping the disease course under surveillance. However, for a miRNA-based therapy it should be kept in mind that a single miRNA can target numerous mRNAs. Consequently, they prevent the translation of numerous proteins that are involved in different cancer-relevant signal transduction pathways. This “multitasking” of miRNAs could also be the reason for the severe adverse effects in patients dosed with MRX34 and that the enrollment and dosing of MRX34 was stopped in the clinical study.

## Conclusion

For the first time, in 2008, the presence of circulating miRNAs in the blood circulation was described, namely in the serum of patients with diffuse large B-cell lymphoma. This was the first evidence showing the high stability of circulating miRNAs in blood and the feasibility of quantifying these molecules^[194,195]^. Regarding the informative content and disease-specific modulation of ncRNAs in the blood of cancer patients, quantification of circulating ncRNAs may deliver new information that can help to understand the mechanisms underlying OC pathogenesis, and to identify new tumor markers as well as therapeutic targets. The eligibility of circulating ncRNAs as biomarkers should cover the dynamic of the disease course, including recurrence and resistance to therapy. For their development, several technical and methodological issues have to be solved. In particular, the choice of an endogenous reference miRNA with constant values is of high relevance, to warrant the reliability of miRNA quantification. The normalization of miRNAs to a reference gene shall eliminate differences in blood sampling, technical handling and RNA quality^[196]^. In addition, the pre-analytical (e.g., serum or plasma, sample preparation) and analytical (e.g., extraction, quantification) aspects of the analyses, as well as the use of different technical platforms should be considered^[197,198]^. All these shortcomings demand for a standardized approach to integrate ncRNAs in future OC detection and treatment. Whole sequencing of ncRNAs along with the establishment of biological network models in which they are involved will provide molecular OC-associated signatures that allow their application for screening or treatment decisions by the physician. The development of such assays will be also beneficial for understanding the pathogenesis of OC.

## Declarations

### Copyright

© The Author(s) 2019.
